# Nanomaterials-Based Electrochemical Δ^9^-THC and CBD Sensors for Chronic Pain

**DOI:** 10.3390/bios13030384

**Published:** 2023-03-14

**Authors:** Dadbeh Pazuki, Raja Ghosh, Matiar M. R. Howlader

**Affiliations:** 1 Department of Electrical and Computer Engineering, McMaster University, 1280 Main Street, Hamilton, ON L8S 4K1, Canada; hajijafd@mcmaster.ca; 2 Department of Chemical Engineering, McMaster University, 1280 Main Street, Hamilton, ON L8S 4LS, Canada; rghosh@mcmaster.ca

**Keywords:** cannabinoids, electrochemical sensing, functionalization, nanomaterials, chronic diseases, cancer painkiller

## Abstract

Chronic pain is now included in the designation of chronic diseases, such as cancer, diabetes, and cardiovascular disease, which can impair quality of life and are major causes of death and disability worldwide. Pain can be treated using cannabinoids such as $\Delta^{9}$-tetrahydrocannabinol ($\Delta^{9}$-THC) and cannabidiol (CBD) due to their wide range of therapeutic benefits, particularly as sedatives, analgesics, neuroprotective agents, or anti-cancer medicines. While little is known about the pharmacokinetics of these compounds, there is increasing interest in the scientific understanding of the benefits and clinical applications of cannabinoids. In this review, we study the use of nanomaterial-based electrochemical sensing for detecting $\Delta^{9}$-THC and CBD. We investigate how nanomaterials can be functionalized to obtain highly sensitive and selective electrochemical sensors for detecting $\Delta^{9}$-THC and CBD. Additionally, we discuss the impacts of sensor pretreatment at fixed potentials and physiochemical parameters of the sensing medium, such as pH, on the electrochemical performance of $\Delta^{9}$-THC and CBD sensors. We believe this review will serve as a guideline for developing $\Delta^{9}$-THC and CBD electrochemical sensors for point-of-care applications.

## 1. Introduction

Chronic diseases (CDs) are non-acute illnesses that are recognized by their long-standing symptoms, such as chronic pain, shortness of breath, and cognitive impairment [1,2]. CDs are major causes of death and disability, reducing the quality of life worldwide [1,2]. In particular, CDs include stroke [1], diabetes [2], cardiovascular diseases [3], chronic obstructive pulmonary diseases [2], and cancer [4]. For example, severe bone pain may be associated with malignant cancer tumors [5,6]. This type of pain is common among cancer patients [7], particularly after chemotherapy, surgery, and radiation therapy [5]. These factors are counted towards health, economic, and social burdens [5]. Despite recent advances in providing highly efficient chronic painkillers, such as opioids, their adverse side effects remain unaddressed [3]. For instance, opioids are both addictive and poisonous [4,8]. Thus, seeking alternative chronic painkillers with minimal side effects is highly demanded.

An alternative approach for alleviating chronic pain can be the use of cannabinoids, such as $\Delta^{9}$-tetrahydrocannabinol ($\Delta^{9}$-THC) and cannabidiol (CBD), which are produced from cannabis plants [3,8]. A small quantity of $\Delta^{9}$-THC has other pharmaceutical benefits, particularly as an anti-inflammatory and neuroprotective agent, while a large quantity of $\Delta^{9}$-THC is psychoactive and addictive [8,9]. On the other hand, a large quantity of CBD is not psychoactive but possesses only pharmaceutical benefits, particularly as a sedative and analgesic [8,10]. Furthermore, purified CBD can alleviate cancer pain, decrease the proliferation of cancer cells, and delay the progression of Alzheimer’s disease [8,10,11]. For example, the Food and Drug Administration has confirmed the efficacy of Epidiolex, a purified CBD, as a robust anti-cancer medicine [12]. Interestingly, the co-administration of CBD with a small amount of $\Delta^{9}$-THC, such as Nabiximols, can act as an effective painkiller for cancer patients [8]. The commercially available Nabiximols contain a fixed ratio of $\Delta^{9}$-THC:CBD (2.7 mg:2.5 mg) in the form of a spray [8,13]. Therefore, frequent monitoring of the concentration of $\Delta^{9}$-THC and CBD in biofluids such as blood and saliva is essential for therapeutic benefits such as pain management [8,10].

Currently, lab-based techniques such as gas chromatography (GC) [14], high-performance liquid chromatography (HPLC) [5], enzyme-linked immunosorbent assay (ELISA) [14], mass spectroscopy (MS) [14], and capillary electrophoresis (CE) [15] are commonly used to accurately detect $\Delta^{9}$-THC and CBD in biofluids [14]. While these methods offer low limits of detection (LOD) and high sensitivity, they are large [5], expensive [6], require trained personnel [14], and involve lengthy measurements [15]. On the other hand, electrochemical sensors, which transduce physical, chemical, or biological parameters into quantifiable electrical signals, have received high attention for detecting Δ^9^-THC and CBD due to their fast and selective detection [16], low LOD [17], and high sensitivity [18,19]. Electrochemical sensors are often low-cost and easier to fabricate [18] and are portable [20] due to their smaller dimensions [17]. Trained personnel are not needed to use the sensors [21]. Furthermore, it is possible to integrate sensors with microelectronic components for real-time monitoring [17,20].

There are many conventional materials, such as polylactic acid (PLA) and conductive polymers, that are utilized for fabricating electrochemical sensors for detecting $\Delta^{9}$-THC and CBD. The maximum concentrations of $\Delta^{9}$-THC and CBD in plasma are on the nanomolar scale [21,22], but the limit of detection (LOD) reported for conventional materials-based electrochemical sensors is at the micromolar scale. The poor sensing performance can originate from the conventional materials’ low electrocatalytic effect and low electrical conductivity.

Nanomaterials, such as nanoparticles, nanotubes, and nanosheets, are used in electrochemical sensors as sensing materials or modifiers. Despite their unique features, such as high electrical conductivity [23] and a large surface area [24], electrochemical sensors based on nanomaterials are less explored than those based on conventional materials like polymers [24]. Combining different types of nanomaterials, such as 0D, 1D, and 2D, can enhance electrochemical signals and detect trace levels of analytes [25]. However, the accuracy, sensitivity, and selectivity of nanomaterial-based electrochemical sensors for $\Delta^{9}$-THC and CBD detection are still lower than those of lab-based techniques [5]. Physicochemical functionalization of the sensors can improve their catalytic activity and lead to the development of new point-of-care (POC) devices for chronic pain management [5]. However, there is a lack of comprehensive studies on this topic from a material science perspective.

In this review, we comprehensively investigate recent advances, research challenges, and future perspectives in applying nanomaterials and conventional materials to develop electrochemical sensors for detecting $\Delta^{9}$-THC and CBD. We study the physicochemical functionalization of nanomaterials compared to conventional materials. Furthermore, we highlight why 2D nanomaterials have better future perspectives compared to 0D or 1D nanomaterials for the electrochemical detection of $\Delta^{9}$-THC and CBD.

## 2. Structures, Oxidations, and Pharmacokinetics of $\Delta^{9}$-THC and CBD

**Figure 1:**
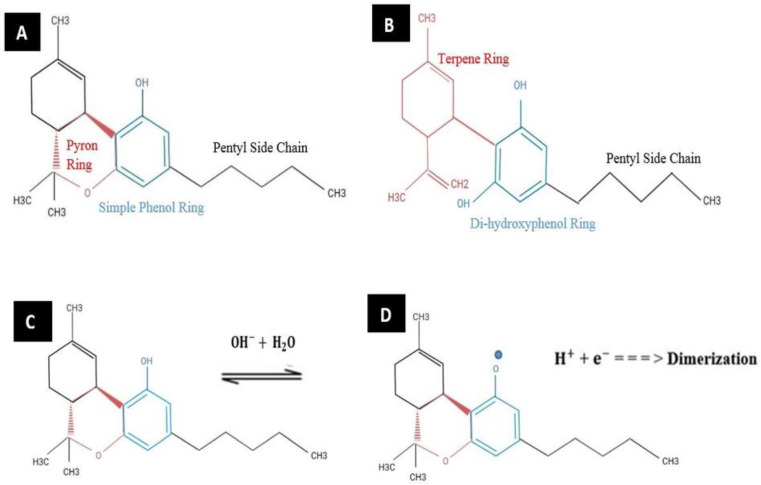
Chemical structures of (**A**) $\Delta^{9}$-tetrahydrocannabinol $(\Delta^{9}$-THC) and (**B**) cannabidiol (CBD). Irreversible oxidation of $\Delta^{9}$-THC: (**C**) before and (**D**) after deprotonation. Reprinted and adapted with permission from ref. [5]. Copyright 2020 American Chemical Society.

Understanding the structures of $\Delta^{9}$-THC and CBD is important for properly analyzing their electrochemical sensing processes. Both cannabinoids contain hydroxyl groups as electroactive groups, making them electrochemically active species [9]. They can be easily detected by common electroanalytical methods, such as cyclic voltammetry and differential pulse voltammetry [11]. Upon oxidation, the hydroxyl groups become deprotonated, leading to the formation of active phenoxy radicals, as shown in Figure 1 [5]. At low potentials, this can result in irreversible dimerization of $\Delta^{9}$-THC and CBD, leading to the formation of an insulating layer on the working electrode surface after the first run [11].

To determine the target sensing characteristics of electrochemical $\Delta^{9}$-THC and CBD sensors for chronic pain management, it is necessary to study the pharmacokinetics of $\Delta^{9}$-THC and CBD. Table 1 summarizes the pharmacokinetic parameters of CBD and $\Delta^{9}$-THC based on their administration routes. $\Delta^{9}$-THC and CBD are present in different biofluids, such as blood and saliva, and depend on the administration route. $\Delta^{9}$-THC is commonly administered through inhalation (smoke and vapor) or oral ingestion [8]. The pharmacokinetic parameters of $\Delta^{9}$-THC vary based on the administration route [26]. If $\Delta^{9}$-THC is administered through inhalation, the majority is absorbed through the lungs [21,26], which leads to fast entry into the bloodstream, quick reach to the brain, and the onset of psychoactive or soothing effects within seconds to a few minutes [21,26]. Smoked $\Delta^{9}$-THC has a *t_max_* of 3–10 min and a *C_max_* of approximately 150 ng/mL, with a bioavailability of 10–35% in plasma [21]. If $\Delta^{9}$-THC is administered through oral ingestion, it undergoes two metabolisms in the liver [26]. In this case, $\Delta^{9}$-THC reaches its maximum concentration of 58 ng/mL in plasma slowly, with a *t_max_* of 1–2 h and even 6 h in some cases [26]. The bioavailability of $\Delta^{9}$-THC is 10–20% [26]. Purified CBD or CBD co-administered with a small amount of $\Delta^{9}$-THC is commonly taken orally [27]. Co-administration of CBD with $\Delta^{9}$-THC does not significantly affect the pharmacokinetics of $\Delta^{9}$-THC [28]. Regardless of the administration route, the bioavailability of CBD is extremely low in plasma [16,23]. Recent human studies show that CBD has a *t_max_* of 1.64–4.2 h and a *C_max_* of 2.05–3.3 ng/mL [16].

## 3. Recent Advances

### 3.1. Direct Electrochemical Detection

#### 3.1.1. Conventional Materials

Here, we investigate recent advances in conventional materials-based electrochemical sensors for detecting $\Delta^{9}$-THC and CBD. Table 2 shows the sensing performance and working-functional materials of conventional material-based electrochemical sensors for detecting $\Delta^{9}$-THC and CBD.

A disposable, low-cost, biodegradable polymeric screen-printed electrode (P-SPE) was used to detect THC [27]. A black conductive polylactic acid (PLA) filament was printed on the base (a brown non-conducting PLA filament) to create a counter, working, and reference electrodes. A non-conducting PLA filament was also printed on the device to separate the electrodes. However, the LOD was 15 µM, which makes the sensor unsuitable for the POC device to detect trace levels of $\Delta^{9}$-THC in plasma [21,26]. The poor LOD was due to PLA’s low electrical conductivity [27]. To improve electrical conductivity and sensitivity, it is possible to create polymeric nanocomposites by mixing PLA filaments with carbon nanomaterials, such as graphene [28,29] and carbon nanotubes [30,31]. This possibility is because carbon nanomaterials have a high electrical conductivity, a large specific surface area, and a significant electrocatalytic effect.

**Figure 2:**
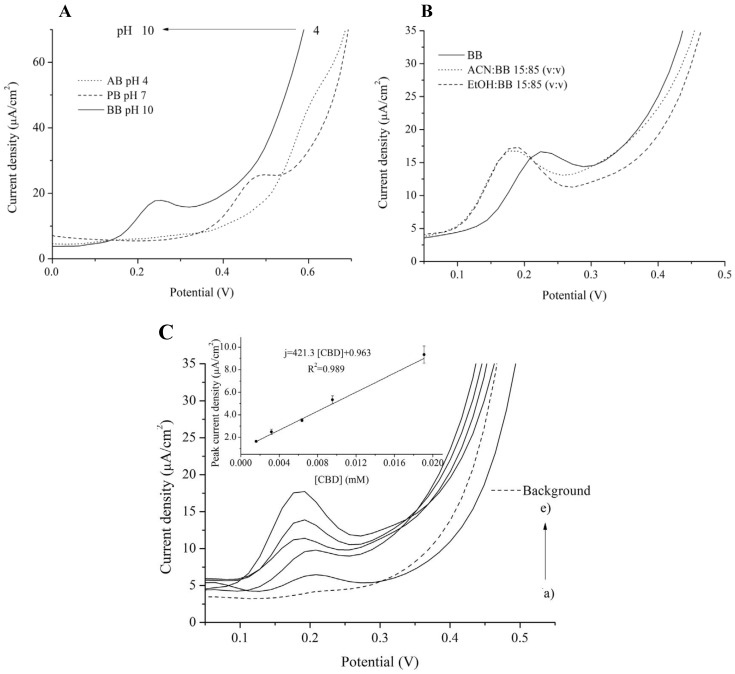
(**A**): DPV signals of Sonogel-Carbon-PEDOT electrodes for 19.1 µM CBD at different pH: acetic or acetate buffer (AB) with pH 4.0, phosphate buffer (PB) with pH 7.0, and boric or borate buffer (BB) with pH 10.0. (**B**): A comparison between DPV signals of Sonogel-Carbon-PEDOT electrodes for 19.1 µM CBD in BB, acetonitrile (ACN):BB (15:85), and ethanol (EtOH):BB (15:85). (**C**): Calibration curve and DPV signals of Sonogel-Carbon-PEDOT electrodes at different CBD concentrations. Reprinted and adapted with permission from ref. [8]. Copyright 2020 Elsevier.

The sonogel route was applied to functionalize carbon paste electrodes to improve the electrochemical detection of CBD [8] The sonogel, a silicon oxide (${SiO}_{2})$ network doped with poly-(3-,4-ethylenedioxythiophene) (PEDOT), created a highly conductive polymeric composite as a modifier. In the final step, graphite powder (the sensing material) was mixed with the modifier. The sonogel route minimized the degradation of PEDOT and increased electron transfer kinetics. Using a higher pH medium (boric or borate buffer (BB) with pH 10.0) produced a sharper oxidation peak at a lower potential (Figure 2A). Adding organic solvents such as ethanol to increase CBD solubility and dispersion in the medium also produced a higher peak at a lower potential (Figure 2B) [10]. The optimized sensor had a linear response (1.59–19.1 µM CBD) (Figure 2C), an LOD of 0.94 µM and a sensitivity of 421 ± 26.1 µA/mM·cm^2^. However, the LOD was not enough to detect trace concentrations of CBD in plasma [16]. This issue was due to the fact that the nanocomposite’s electrical conductivity and electrocatalytic effect were not high enough. To address this issue, it is possible to dope ${SiO}_{2}$ with conductive polymers, such as polyaniline (PANI), and coat it with nanomaterials, such as gold nanoparticles (Au NPs) [32], which may lead to a lower LOD.

**Figure 3:**
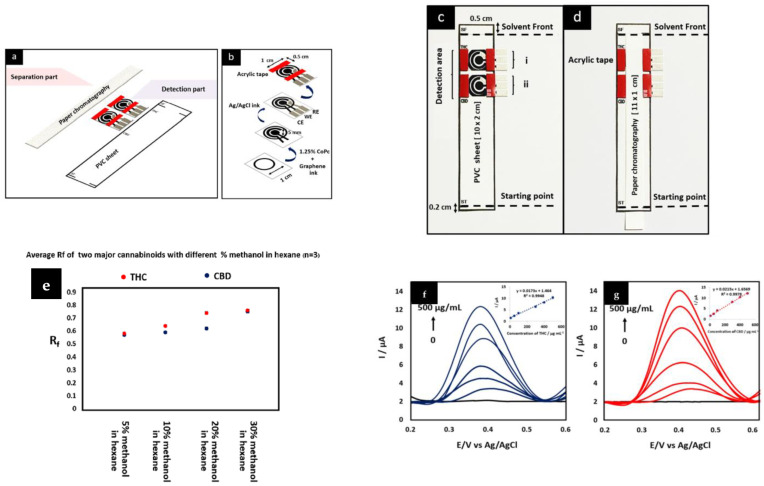
(**a**) Three major parts of the device, including the PVC sheet (substrate), modified screen printed electrodes (detection part), and chromatographic paper (separation part). (**b**) Functionalization process for modifying graphene ink with 1.25% cobalt phthalocyanine (CoPc) and printing Ag or AgCl ink on the substrate for fabricating reference electrode. (**c**) The device after mounting the detection part and (**d**) the separation part on the PVC sheet. (**e**) Different methanol concentrations in hexane and their influence on the amount of retardation factors ($R_{f}$) of CBD (black spots), $\Delta^{9}$-THC (red spots) in the mobile phase, and DPV signals. The calibration curves of (**f**)$\Delta^{9}$-THC and (**g**) CBD were obtained from CoPc modified-screen printed graphene electrode (CoPc/SPGE) in 0.1 M PBS with pH 7.0. Reprinted and adapted with permission from ref. [7]. Copyright 2022 Elsevier.

A chromatographic paper-based electrochemical device was used to simultaneously detect $\Delta^{9}$-THC and CBD in cannabis oil (Figure 3a–d) [7]. The separation between $\Delta^{9}$-THC and CBD in the oil was based on their retardation factors (Rf) (Figure 3e). To improve the DPV signals, graphene ink (as the sensing material) was mixed with cobalt phthalocyanine (CoPc, a metallic complex modifier) for physical functionalization. The hybrid CoPc and graphene ink electrocatalytic effects resulted in approximately 2- and 1.5-fold increases in peak currents for $\Delta^{9}$-THC and CBD, respectively. The device showed a linear response at a concentration range of 10–500 µg/mL for both cannabinoids (Figure 3f–g). The sensitivity was 0.0215 µA·mL/µg and 0.0173 µA·mL/µg for CBD and $\Delta^{9}$-THC, respectively. Additionally, the LODs were 3.27 µg/mL and 2.85 µg/mL for $\Delta^{9}$-THC and CBD, respectively. Notably, while the time required to separate both cannabinoids in the cannabis oil was approximately one-fifth that of ultra-high-pressure LC-MS (UPLC-MS), the LOD was higher than that of UPLC-MS [7] The LOD can be improved by using nanocomposites made of graphene ink, CoPc, and carbon nanomaterials, such as MWCNTs [33] and carbon black NPs [34]. This usage can strengthen the hybrid electrocatalytic effect due to the intrinsic carbon nanomaterials’ features [33,34].

An aptamer-modified gold SPE (G-SPE) was developed for detecting $\Delta^{9}$-THC in phosphate buffered saline (PBS) and saliva [35]. The modified G-SPE was combined with a microfluidic system consisting of a polydimethylsiloxane channel, sample collector, and filtering system and connected to a smartphone for data visualization. The bare electrode did not require signal amplification due to gold’s high electrocatalytic effect and electrical conductivity. The sensor was able to detect $\Delta^{9}$-THC in the presence of CBD and cannabinol (CBN) thanks to the aptamers’ high affinity for $\Delta^{9}$-THC. In PBS, the LOD was 1 nM, while in saliva it was ten times higher due to interfering elements. The aptamer sensor had adequate storage ability, remaining stable for up to 3 days at 4 °C, and was reusable for up to 5 cycles by washing the electrode with PBS.

**Table 2:** A comparison between conventional material-based electrochemical sensors for detecting $\Delta^{9}$-THC and CBD in terms of their sensing performance and working-functional materials.

Electrode	Technique	Sensitivity	LOD (ng/mL)	LR (ng/mL)	Selectivity	Ref.
Black Conductive PLA	DPV	---------	4717.03 (PBS)	--------	--------	[27]
Silicon Oxide/doped PEDOT/CPE	DPV	421 ± 26.1 µA/mM.cm^2^	295.6 (ACN:BB) (15:85) (EtOH:BB) (15:85)	185.52–6005.1	---------	[8]
CoPc/SPE	DPV	173 × $10^{-7}$ µA·mL/ng 215 × $10^{-7}$ µA·mL/ng	3270 2850 (PBS)	$10^{4}$–5 × $10^{5}$	Paper Chromotography	[7]
Aptamer/G-SPE	DPV	---------	0.314 (PBS) 3.14 (Saliva)	---------	Aptamer	[35]
MIP/NSC/GCE	DPV	--------	0.91 (Cannabis oil)	1.26–25.16 × $10^{4}$	MIP	[36]

An electrochemical sensor using ratiometric measurement was developed to detect CBD in both cannabis oil and human serum samples [36]. The sensor was composed of a GCE (bare electrode), nitrogen and sulfur co-doped carbon (NSC) materials (as functional materials), and multifunctional MIPs (receptor). NSC was produced through the pyrolysis of Azura A and was drop-casted on the GCE to increase the electrocatalytic effect and provide large active sites for MIP film formation. Multifunctional MIPs were created with Fe (as the doped active center), aminophenanthroline (AP, as the monomer), and 3,4-ethylene dioxythiophene (EDOT, as another monomer). MIP thin films were deposited on the NSC using the electropdeposition method. Fe acted as an active center, resulting in better electrocatalytic activity for the MIP and a constant electrooxidation signal (internal reference signal) for CBD ratiometric electrochemical sensing. The sensor showed a linear response to a wide range of CBD concentrations (0.004–0.8 µM), with a LOD of 2.9 nM. The sensor also exhibited robust selectivity in detecting CBD in the presence of uric acid (UA), dopamine (DA), glucose (Glu), and urea. The sensing performance was evaluated in human serum samples, with a recovery percentage of 97.2–114%, confirming the outstanding sensing performance of the sensor in real-world applications.

The maximum concentration of CBD and $\Delta^{9}$-THC in plasma is in the nanomolar range, as shown in Table 1 [19,21,26]. The last two sensors [35,36] in Table 2 were only able to detect at the nanomolar scale, indicating their capability to detect $\Delta^{9}$-THC in the plasma of patients with chronic pain.

#### 3.1.2. 0D Nanomaterials

The use of nanoparticles (NPs) as 0D nanomaterials to improve the performance of electrochemical sensors has received significant attention in recent years [27]. NPs have unique physicochemical properties that are less commonly explored in conventional materials [22]. These properties include a high surface-to-volume ratio [13], exceptional electron transfer kinetics [36], and good adsorption ability [22]. According to the literature, three nanoparticle-modified electrochemical sensors have been developed for the detection of cannabinoids. Table 3 shows a comparison of the sensing performance and working-functional materials of nanoparticle-modified electrochemical sensors detecting $\Delta^{9}$-THC and CBD.

An electrochemical immunosensor that is disposable was developed for the simultaneous detection of morphine (MOR), $\Delta^{9}$-THC, and benzoylecgonine (BZC) in PBS and urine [28]. To improve sensitivity, a disposable electrically printed (DEP) carbon electrode was modified with gold nanoparticles (Au NPs), amplifying the sensitivity of the bare electrode due to their high electrocatalytic effect and high surface-to-volume ratio. To ensure robust selectivity, corresponding antibodies were used. Cysteamine and glutaraldehyde were employed as mediators to covalently attach the corresponding antibodies to the working electrode, creating specific binding sites for each target analyte on the working electrodes. To evaluate the antibody performance, bovine serum albumin (BSA) was also embedded on the working electrodes as a competitive receptor. The corresponding antibodies successfully detected target analytes in the presence of BSA. The LOD for detecting $\Delta^{9}$-THC was 7.0 pg·${mL}^{-1}$, and the immunosensor had a linear response within the $\Delta^{9}$-THC concentration range of 10.0 pg·${mL}^{-1}$ to 10.0 µg·${mL}^{-1}$. Interestingly, the immunosensor’s LOD and selectivity were comparable to those of commercial ELISA kits, while the immunosensor’s fabrication cost was significantly lower than that of the ELISA kits. The recovery percentage was in the range of 88.0–115.1% for detecting $\Delta^{9}$-THC in urine, confirming the outstanding sensing performance for analyzing $\Delta^{9}$-THC trace concentrations in real samples. However, the sensor’s selectivity could be affected by temperature fluctuations [28], as changes in antibody binding constants may occur due to temperature variations [37]. The utilization of aptamers may address the issue owing to their high thermal stability [38,39].

**Table 3:** A comparison of the sensing characteristics and functional materials of nanoparticle-modified electrochemical sensors for detecting $\Delta^{9}$-THC and CBD.

Electrode	Technique	Sensitivity (µA·mL/ng)	LOD (ng/mL)	LR (ng/mL)	Selectivity	Ref.
antibody/cys/glu/AuNPs/DEP	SWV	--------	0.007 (PBS)	0.01–10,000.0	Antibody	[28]
CBNPS/GCE	CV, and DPV	0.206 × $10^{-3}$	110 (cannabis oil)	300.0–2000.0	--------------	[40]

A drop-casted carbon black nanoparticle (CBNP)/glassy carbon electrode (GCE) was utilized to detect CBD in cannabis oil [40]. CBNPs amplified GCE signals due to their high surface-to-volume ratio and electrocatalytic effect. In addition to GCE functionalization, three approaches were also followed to achieve stronger signals. Firstly, the working electrode was polarized at a fixed potential of 0.7 V, which facilitated the transfer of CBD to the working electrode before conducting electrochemical measurements. Secondly, acetonitrile was added to the Briton Robinson buffer to increase the solubility and dispersion of CBD in the medium. Lastly, the pH of the medium was increased from 7.0 to 10.0, which also contributed to signal amplification through the increased diffusion rate of CBD. Under optimized conditions, the sensor linearly responded to CBD concentrations in the range of 0.3–2.0 mg/L. The LOD and sensitivity were 0.11 mg/L (0.35 µM) and 0.206 µA·L·${mg}^{-1}$, respectively. Despite CBNPs’ high electrocatalytic effect, the LOD was not desirable for detecting CBD in the plasma of patients with chronic pain [16]. Functionalization of CBNPs with cetrimonium bromide (CTAB, receptor) can simultaneously improve sensitivity and selectivity [40]. This can originate from the robust interactions between CTAB’s hydrophobic tail (long alkyl chain) and CBD’s di-hydroxyphenol ring (electroactive group).

As previously mentioned, the maximum concentration of CBD and $\Delta^{9}$-THC in plasma is on the nanomolar scale [16,21,26]. Among the nanoparticle-modified electrochemical sensors mentioned, the first one can only detect at the picomolar scale [28].Therefore, this sensor is highly sensitive and desirable for detecting $\Delta^{9}$-THC in the plasma of patients suffering from chronic pain.

#### 3.1.3. 1D Nanomaterials

Multi-walled carbon nanotubes (MWCNTs), as one-dimensional nanomaterials, have been widely used to improve the performance of electrochemical sensors. MWCNTs possess unique physicochemical properties, including a high surface-to-volume ratio [41], excellent electron transfer kinetics [42], good adsorption ability [13], excellent mechanical stiffness [43], and good functionalization ability [44], which are not as well explored in conventional materials. To the best of our knowledge, only one MWCNTs-modified electrochemical sensor has been introduced for the detection of $\Delta^{9}$-THC. Table 4 shows the working electrode materials and sensing performance.

**Figure 4:**
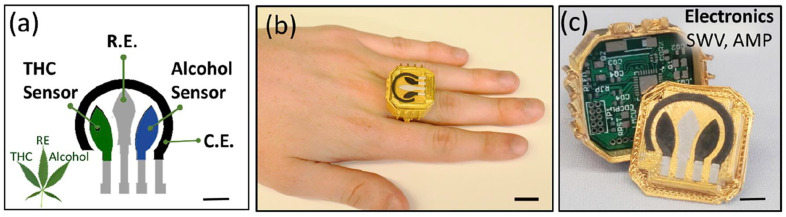
(**a**): The leaf-like sensor configuration, including dual working electrodes with the same counter electrode (C.E.) and reference electrode (R.E.). (**b**): Wearable electrochemical ring sensor. (**c**): The case, electronic board, and sensing system. Reprinted and adapted from ref. [45]. Copyright 2020 Elsevier.

An innovative wearable ring sensor was developed for the simultaneous detection of $\Delta^{9}$-THC and alcohol in PBS as well as diluted saliva (Figure 4a–c) [45]. The sensor operated based on dual working electrodes with the same reference and counter electrode. The alcohol electrode was modified with Prussian-blue (PB) and functionalized with alcohol oxidase or chitosan (CH) or glutaraldehyde (GLU). The $\Delta^{9}$-THC electrode was a modified graphite electrode mixed with 1% MWCNTs for improved sensitivity. The sensor responded linearly to 1–6 µM $\Delta^{9}$-THC in 0.1 M PBS at pH 7.0 with a LOD of 0.5 µM. However, the sensor was not suitable for detecting nanomolar concentrations of $\Delta^{9}$-THC in the plasma [21,26]. MWCNTs can be chemically functionalized with metal or metal oxide NPs, e.g., Au NPs and NiO NPs, respectively [46,47]. This results in a synergistic electrocatalytic effect, higher electrical conductivity, and lower LOD acquisition. The data were interpreted using Matlab [45]. The utilization of machine learning and artificial intelligence (AI) is proposed to carry out more accurate data analysis [48].

#### 3.1.4. 2D Nanomaterials and Nanocomposites

As previously mentioned, 2D nanomaterials have a high specific surface area [15], great mechanical stiffness [48], good functionalization ability [13], high electrical conductivity [15], and a strong electrocatalytic effect [13]. Currently, nanocomposites made of graphene/MWCNTs as the matrix and metal oxide NPs or conductive polymers as the second component are being used for direct electrochemical detection of cannabinoids. To the best of our knowledge, three sensors using nanocomposite-modified carbon electrodes have been developed to detect $\Delta^{9}$-THC and CBD [49,50,51]. Table 5 compares the sensing performance and materials used in these sensors.

**Figure 5:**
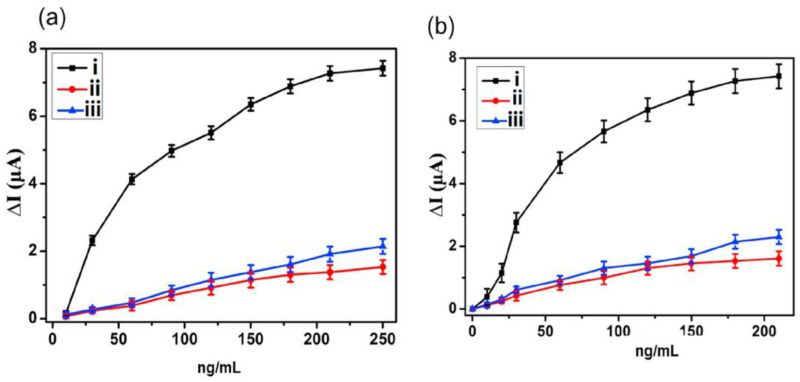
ΔI as a function of i: $\Delta^{9}$-THC, ii: caffeine, and iii: acetaminophen utilizing (**a**): carbon beads/MIPs electrode and (**b**): CNT/MIP electrode. Reprinted and adapted with permission from ref. [50]. Copyright 2019 Elsevier.

In the first sensor, a molecularly imprinted carbon electrode was introduced for detecting $\Delta^{9}$-THC in a 1:1 methanol: deionized water mixture [50]. Two sensing materials, carbon nanotubes, and carbon beads were used. Poly (methyl acrylic acid-co-ethylene glycol dimethacrylate) was used as the MIP to achieve robust selectivity. The sensing materials were doped within the MIP matrix, which was copolymerized at 70 °C. The working electrode was then immersed in methanol for 24 h to remove the template ($\Delta^{9}$-THC). The same process was used for the non-imprinted polymer (NIP), except for the removal of the template. Both sensors could detect $\Delta^{9}$-THC at the nanomolar scale due to the high electrocatalytic effect and electrical conductivity of the sensing materials. The MIP/CNT electrode showed slightly better sensitivity due to the higher surface-to-volume ratio of CNTs, with an LOD of 0.32 ± 0.02 ng/mL. The LOD for the MIP/carbon beads electrode was 0.18 ± 0.02 ng/mL. Both sensors were highly selective for $\Delta^{9}$-THC in the presence of caffeine (CAF) and acetaminophen, as the cavities inside the MIPs had a high affinity for $\Delta^{9}$-THC (Figure 5).

The second sensor improved the detection of CBD by functionalizing a GCE with a nanocomposite of amino iron oxide nanoparticles and graphene [51]. The graphene and Fe_3_O_4_ nanoparticles amplified the electrochemical signals due to their hybrid electrocatalytic effects. The attached amino groups on the Fe_3_O_4_ nanoparticles also strengthened the signals by decreasing the GCE water angle and enhancing CBD adsorption on the working electrode. The LOD was 0.04 µM, and the sensor had a wide dynamic range with three different linear ranges. At scan rates between 5 mV/s and 200 mV/s, CBD oxidation was controlled by adsorption, and there was a linear relationship between peak current and scan rate. However, at scan rates above 200 mV/s, CBD oxidation involved both adsorption and diffusion, and there was a linear relationship between the logarithm of peak current and the logarithm of scan rate.

**Figure 6:**
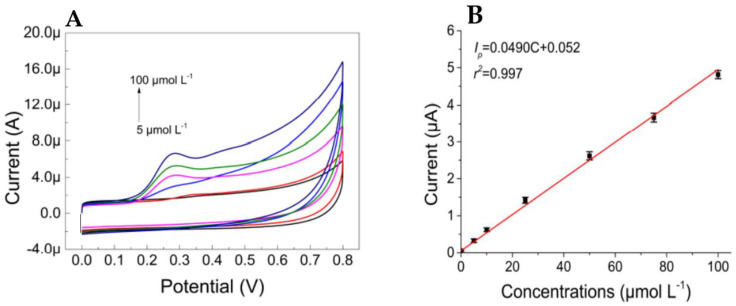
(**A**): CV responses from mag–MIP/Gr–UiO66/SPE at different CBD concentrations and (**B**): the linear relationship between peak current and CBD concentration. Reprinted and adapted from ref. [52]. Copyright 2022 Multidisciplinary Digital Publishing Institute (MDPI).

In the last study, a modified carbon SPE (C-SPE) made of UiO66/graphene/mag-MIP/carbon was presented for detecting CBD in PBS and cannabis oil [52]. In the first step, a graphene-UiO66 nanocomposite was drop-cast onto the C-SPE, which physically functionalized it. UiO66 was a metal–organic framework consisting of zirconium oxide ions and 2-aminoterephthalate ligands. The nanocomposite enhanced the electrode sensitivity due to the hybrid electrocatalytic effect of graphene and UiO66. UiO66’s porous structure also enabled the selective detection of CBD in the presence of other cannabinoids like $\Delta^{9}$-THC. To improve selectivity, the graphene/UiO66/C-SPE was modified with magnetic MIP (mag-MIP) by drop-casting. The MIP matrix contained Fe_3_O_4_ NPS as the core and CBD-adsorbing cavities (Figure 6B). The mag-MIP was formed by polymerizing methyl acrylic acid and Fe_3_O_4_ NPS at 60 °C. The magnetite nanoparticles increased the MIP matrix’s electrical conductivity and stability. The LOD and sensitivity were 0.05 µM and 0.0490 µA·µ$M^{-1}$, respectively. The sensor linearly responded to CBD concentrations of 5–100.0 µM, with oxidation controlled by adsorption and a linear relationship between peak current and scan rate. The recovery percentage was 99.5% to 99.8%, demonstrating excellent performance in detecting CBD in cannabis oil.

All sensors discussed above had the capability to detect $\Delta^{9}$-THC and CBD in the plasma of chronic pain patients [16,21,26]. They can be considered adequate POC devices.

### 3.2. Alternative Sensors

Direct electrochemical detection is not the only method used to characterize $\Delta^{9}$-THC in biofluids or synthetic buffer solutions. In recent years, alternative sensors, such as organic electrochemical transistors, have been utilized to detect target cannabinoids on the nanomolar or picomolar scale. Table 6 lists these sensors in terms of working electrode materials, sensing mechanisms, and sensing performance.

Four recent papers have described methods for detecting the presence of $\Delta^{9}$-THC in saliva and urine. The first method used giant magnetoresistive (GMR) biosensors [53]. The system included a printed circuit board, Bluetooth module, and smartphone and measured changes in resistance caused by the oxidation of $\Delta^{9}$-THC. Higher concentrations resulted in lower resistance. The GMR biosensor chip was made by depositing IrMn, CoFe, Ru, Cu, and CoFe on a silicon wafer and immobilizing antibodies and BSA for selectivity. The GMR biosensor can detect $\Delta^{9}$-THC concentrations in the range of 0–200 ng/mL (dynamic range (DR)). The system is fast and portable, but its sensitivity to temperature variations is a big drawback.

The second method described involves using a flexural plate-wave-based micro-electromechanical system biosensor to detect THC in urine [54]. The system consisted of a biosensor, an FPGA board, an ARM board, and a battery. Waves with different frequencies were generated based on THC oxidation, with a linear relationship between frequency shift and THC concentration. The biosensor had a transducer with SiO_2_, Si_3_N_4_, Cr, Au, and ZnO films and a receptor with an etched Si wafer, Cr, Au films, and THC antibodies. It had high sensitivity, fast response, and selectivity but was affected by temperature fluctuations. The LOD and linear range were 1.5625 ng/mL and 1.5625–50 ng/mL, respectively.

The two sensors described have the capability to detect $\Delta^{9}$-THC in the plasma of patients with chronic pain [21,26]. However, as previously mentioned, temperature variations can affect the antibody binding constant [37]. Immobilization of MIPs/aptamers on transducers can address this issue and provide comparable selectivity. This is due to the high thermal stability and significant affinity of the MIP/aptamer binding sites with the target analyte [22,55].

**Table 6:** A comparison between alternative sensors for detecting $\Delta^{9}$-THC in terms of working electrode material, sensing mechanism, and sensing performance.

Electrode	Sensing Mechanism	Sensitivity	LOD (ng/mL)	LR-DR (ng/mL)	Selectivity	Ref.
IrMn/CoFe/Ru/Cu/CoFe/Silicon Wafer/Antibody/BSA	Resistance Changes due to THC oxidation	----------	----------	0–200	Antibody	[53]
Si$O_{2}$ **/** ${Si}_{3}N_{4}$/Cr/Au/Zn/Etched Silicon Wafer/Cr/Au/Antibody	Shifts Frequency due to THC Oxidation	-----------	1.5625 (Urine)	1.5625–50	Antibody	[54]
Platinum	Changes in bias between source and drain due to THC oxidation	0.0162 ± 0.003/dec(DI water) −0.126 ± 0.004/dec(DI water) −0.003/dec (saliva) −0.02/dec (saliva)	0.031 (DI water) 0.31 (saliva)	0.031–180.2 (DI water) 180.2–1572.35 (DI water) 0.31–133.65 (saliva) 133.65–1572.35 (saliva)	-------	[56]
NanoMIP/Gold	Capacitance changes due to THC oxidation	-----------	0.31 × $10^{-6}$ (PBS)	0.31 × $10^{-3}$–3144.7	MIP	[57]

The third method described involves using Organic Electrochemical Transistors (OECTs) to detect $\Delta^{9}$-THC by measuring changes in a constant bias between the gate and drain [56]. OECTs were used to detect $\Delta^{9}$-THC in deionized water (DI) and synthetic saliva buffers using an aerosol jet printing technique. The OECTs consisted of a silver source or drain, PEDOT: polystyrene sulfonate as channel materials, and a platinum gate. The oxidation of $\Delta^{9}$-THC changed the constant bias between the silver gate and drain, with an inverse relationship between the drain current value and $\Delta^{9}$-THC concentration. The platinum gate did not require signal amplification due to its high conductivity and electrocatalytic effect. Detection was done using cyclic voltammetry, with limits of detection of 0.1 nM in DI water and 1 nM in synthetic saliva buffer. The sensor can be considered an adequate POC device owing to its high sensitivity at the nanomolar scale and fast detection time. However, poor selectivity was a big drawback. Immobilization of MIPs/aptamers on platinum can significantly improve selectivity. This is due to the high affinity of the MIP/aptamer binding sites with the target analyte [22,55].

The fourth method described involves using gold capacitive sensors to detect $\Delta^{9}$-THC in PBS [57]. NanoMIPs (receptors) were deposited on the surface of the gold capacitive sensors (transducers). To create the nanoMIPs, six monomers, including acrylamide were used and polymerized under UV radiation for 1 min and 30 s. Binding $\Delta^{9}$-THC to the receptor caused changes in capacitance (ΔC), forming a reciprocal relationship with the logarithm of $\Delta^{9}$-THC concentration. With a high surface-to-volume ratio and affinity for $\Delta^{9}$-THC, the sensor had both high sensitivity and selectivity. It showed a linear response at $\Delta^{9}$-THC concentrations of 0.01 pM to 10 µM, with a LOD of 0.01 pM. The sensor had a fast response time (45 min), a simple detection method, and required small sample volumes. With its low LOD and robust selectivity, this sensor can be considered a POC device for detecting $\Delta^{9}$-THC in the plasma of patients with chronic pain [21,26].

## 4. Research Challenges and Future Perspectives

There are still challenges in using $\Delta^{9}$-THC and CBD electrochemical sensors as reliable point-of-care devices for managing chronic pain.

### 4.1. Sensitivity

The sensitivity of most fabricated $\Delta^{9}$-THC and CBD electrochemical sensors is lower compared to laboratory-based techniques such as liquid chromatography/mass spectroscopy and other types of electrochemical sensors like GMR biosensors. To achieve comparable sensitivity, two approaches are proposed. Firstly, utilizing 2D nanomaterials as transducers directly shows promise due to their outstanding electrocatalytic effect, high electrical conductivity, mechanical stiffness, and high surface-to-volume ratio. Secondly, immobilizing surfactants, such as CTAB, on the working electrode may lead to a decrease in its wetting angle and an increase in the adsorption of target cannabinoids to the working electrode. This factor is because surfactants are amphiphilic compounds [57]. In addition to potential sensitivity improvement, using surfactants is a simple [57], cost-effective [58], and environmentally friendly functionalization approach [59].

### 4.2. Selectivity

$\Delta^{9}$-THC and CBD are irreversibly oxidized at the same potential, which leads to overlapping oxidation peaks when both are detected simultaneously [5,6]. To address this issue, the use of antibodies, MIPs, and metal–organic frameworks have been introduced [27,49,51]. Among these, the use of antibodies resulted in the most robust selectivity in complex media and a low detection limit at the picomolar scale [27]. However, the use of antibodies has limitations, such as difficulty in immobilizing well-oriented antibodies on the sensor and changes in device selectivity performance due to changes in temperature [5,21,51]. Additionally, antibodies may adsorb similar species instead of $\Delta^{9}$-THC and CBD in the sample, resulting in false positive results [16]. A promising alternative is the use of aptamers. Aptamers have comparable selectivity capability to immunosensors while being more thermally and chemically stable against medium ionic strength and temperature [60,61]. Synthesizing aptamers is also more cost-effective, easier, and less time-consuming than synthesizing antibodies, and aptamer-based sensors can be easily reused after following washing protocols. Therefore, to overcome the limitations of antibody-based electrochemical sensors, the use of aptamers is a promising option for achieving robust selectivity.

### 4.3. Surface Fouling

Once $\Delta^{9}$-THC and CBD are oxidized, an insulating layer can form on the working electrode [10]. This phenomenon, known as surface fouling, is caused by the dimerization of neighboring phenoxy radicals near the working electrode [7]. This reduces the number of electroactive sites on the electrode and decreases its sensing capability for further electrochemical measurements [7]. As a result, cannabinoid electrochemical sensors are typically disposable or require surface renewal after the first measurement [7]. While mechanical polishing has been suggested as a simple and efficient method of removing the insulating layer from certain types of working electrodes, such as GCEs [7], finding a material that can effectively prevent the formation of the insulating layer is a key research challenge.

### 4.4. Real-Time Monitoring

Wearable electrochemical sensors for $\Delta^{9}$-THC and CBD require further research and development to create POC devices. These devices typically require sensing electrodes, microfluidic and filtering systems, electronics, and wireless communication for data transmission [62,63,64,65,66]. However, integrating all of these components to create small, non-invasive platforms is not easy [62,65,66,67,68]. Detecting cannabinoids in biofluids, such as saliva, tears, and subcutaneous interstitial fluid non-invasively is a challenge [47,69]. Additionally, biofluids contain other components that may interfere with the detection of cannabinoids, reducing sensitivity [70]. Data processing using advanced machine learning and AI is also challenging, particularly for predicting chronic diseases [47,69]. For chronic pain management, data scientists and healthcare professionals not only need to analyze and interpret the data for $\Delta^{9}$-THC and CBD [47,71], but also other biomarkers such as glutamate [72,73] and interleukin-6 [74], which are associated with chronic pain. Overall, there is significant potential for creating wearable electrochemical $\Delta^{9}$-THC and CBD sensors.

## 5. Conclusions

Many individuals worldwide, from adults to the elderly, suffer from chronic pain. Administration of purified CBD or co-administration of CBD with a small quantity of $\Delta^{9}$-THC has shown promise in alleviating chronic pain due to its minimal side effects. In recent years, nanomaterial-based electrochemical sensors for detecting $\Delta^{9}$-THC and CBD have gained attention for their fast response, low cost, and user-friendliness. However, the accuracy, sensitivity, and selectivity of most nanomaterial-based electrochemical sensors for $\Delta^{9}$-THC and CBD detection are still lower than those of lab-based techniques and other types of electrochemical sensors. To improve sensitivity, two approaches have been proposed: the direct use of 2D nanomaterials as the sensing material due to their outstanding electrocatalytic effects, great mechanical stiffness, and high-surface-to-volume ratio, and the functionalization of the working electrodes by surfactants to decrease the water contact angle of the active sensor surface.

To improve selectivity, the use of aptamers and MIPs have been suggested due to their cost-effectiveness, simplicity, and less time-consuming preparation methods compared to antibodies. To the best of our knowledge, no one has directly used 2D nanomaterials, surfactants, and aptamers to improve the electrochemical measurements of cannabinoids. If the limitations regarding sensitivity, selectivity, surface fouling, and real-time monitoring of electrochemical sensors detecting $\Delta^{9}$-THC and CBD are addressed, we will witness a new type of POC device that would be beneficial for gaining insights into the concentration of $\Delta^{9}$-THC and CBD in plasma and saliva, thus alleviating chronic pain in the foreseeable future.

## Figures and Tables

**Table 1:** The pharmacokinetic parameters of CBD and $\Delta^{9}$-THC.

Cannabinoid	Administration Route	Bioavailability in Plasma	$t_{max}$	$C_{max}$	Ref.
CBD	Oral	Little to none	1.64–4.2 h	2.05–3.3 ng/mL	[16]
$\Delta^{9}$-THC	Inhalation (smoking)	10–35%	3–10 min	150 ng/mL	[21]
$\Delta^{9}$-THC	Oral	10–20%	1–2 h or even 6 h	58 ng/mL	[26]

**Table 4:** An MWCNTs-modified ring electrochemical sensor for detecting $\Delta^{9}$-THC was developed, along with its sensing characteristics and working-functional materials.

Electrode	Technique	Sensitivity (µA·mL/ng)	LOD (ng/mL)	LR (ng/mL)	Selectivity	Ref.
WE1: 1% MWCNTs/Graphite Ink WE2:alcohol oxidase/CH/GLU/PB ink	SWV CA	--------	157.23 PBS	314.47–18886.82	Dual Working Electrode	[45]

**Table 5:** A comparison between nanocomposite-modified electrochemical sensors for detecting $\Delta^{9}$-THC and CBD in terms of their sensing characteristics and working-functional materials.

Electrode	Technique	Sensitivity (µA·mL/ng)	LOD (ng/mL)	LR (ng/mL)	Selectivity	Ref.
CNT/MIP	DPV	------------	0.18 ± 0.02 Methanol: DIW (1:1)	-----------	MIP	[50]
${NH}_{2}$-${Fe}_{3}O_{4}$NPs/GN/GCE	CV	4.08 × $10^{-3}$ 0.56 × $10^{-3}$ 0.19 × $10^{-3}$	12.58 (PBS)	31.45–306.29 306.29–6130.28 6130.28–314,470.0	---------	[51]
MagMIP/graphene/UiO66/SPE	CV	0.16 × $10^{-6}$	15.72 (PBS)	1572.35–314,470.0	MIP	[52]

## Data Availability

Not applicable.
